# Search for Human Lactate Dehydrogenase A Inhibitors Using Structure-Based Modeling

**Published:** 2015

**Authors:** D. K. Nilov, E. A. Prokhorova, V. K. Švedas

**Affiliations:** Belozersky Institute of Physico-Chemical Biology, Lomonosov Moscow State University, Leninskie Gory 1, bldg. 40, 119991 Moscow, Russia; Faculty of Bioengineering and Bioinformatics, Lomonosov Moscow State University, Leninskie Gory 1, bldg. 73, 119991, Moscow, Russia

**Keywords:** docking, inhibitor, lactate dehydrogenase, molecular modeling

## Abstract

The human lactate dehydrogenase isoform A plays an important role in the anaerobic metabolism of tumour cells and therefore constitutes an attractive target in the oncology field. Full-atom models of lactate dehydrogenase A (in complex with NADH and in the apo form) have been generated to enable structure-based design of novel inhibitors competing with pyruvate and NADH. The structural criteria for the selection of potential inhibitors were established, and virtual screening of a library of low-molecular-weight compounds was performed. A potential inhibitor, STK381370, was identified whose docking pose was stabilized through additional interactions with the loop 96-111 providing for the transition from the open to the closed conformation.

## INTRODUCTION

Lactate dehydrogenase (LDH) catalyzes the NADH-driven conversion of pyruvate to lactate at the final stage of anaerobic glycolysis. In view of tumor energy metabolism, involving glycolysis activation and inhibition of respiratory chain activity (known as the Warburg effect) [1], human LDH has emerged as a promising tumor promoting factor and a therapeutic target. Glycolic rates in tumor cells could be elevated by an increased level of lactate dehydrogenase isoform A (LDH-A) [2, 3]. Thus, selective inhibition of LDH-A can arrest ATP production and promote tumor cell death [4-6]. Another point to bear in mind is distinguishing between LDH-A and LDH-B (heart muscle LDH) that exhibit high structural similarity [7]. Available X-ray structures of human LDH-A, as well as knowledge of the active site configuration and the catalytic mechanism, provide a means for discovery and structural optimization of inhibitors.

LDH-A is comprised of four subunits, each of which has an active site. Initial binding of the coenzyme NADH by subunit is followed by binding of pyruvate. This is mediated by the Arg168 side chain that forms twin hydrogen bonds with the carboxyl group of pyruvate [8]. In the reaction mechanism hydride ion is transferred to the carbonyl carbon of pyruvate from NADH and proton is donated to the carbonyl oxygen from His192. The loop 96-111 is essential for catalysis, closing over the active site of LDH-A after the coenzyme and substrate are bound. Being the rate-limiting step, loop closure favors hydrogen bond formation between pyruvate and Arg105 to stabilize the transition state [9]. The structure of human LDH-A crystallized as a ternary complex in the presence of NADH and oxamate (PDB ID 1i10) shows that transition of the loop 96-111 from the open to the closed form may not necessarily occur following substrate binding [7]. Two of the eight subunits remain in the open conformation in the asymmetric units (D and G). A recent study of the crystal structures of the apo form and NADH binary complexes of human LDH-A (PDB ID 4l4r and 4l4s, respectively) demonstrated that the binding of NADH only induces small-scale local changes in the loop structure [10].

Despite a great deal of research into the structural and physico-chemical properties of LDH-A, only a few classes of LDH-A inhibitors have been described, with most compounds having low potencies [11]. The reference substrate-like inhibitor of LDH is oxamate, with a dissociation constant of 26 μM against human LDH-A [12]. N-substituted oxamates also inhibit different LDH isoforms in the micromolar range [13, 14]. Recently, AstraZeneca and ARIAD Pharmaceuticals unveiled new LDH-A inhibitors: derivatives of malonic and nicotinic acids [15, 16]. These compounds were obtained by linking of molecular fragments recognized by the substrate-binding and coenzyme-binding sites. These fragments were identified using high-throughput screening of compound databases, involving molecular modeling at certain points. A crystal structure of human LDH-A in complex with one of the most efficient inhibitors (PDB ID 4ajp) was determined, with the loop 96–111 in the closed conformation. Interestingly, the effective binding does not require loop transition to the closed form, since several enzyme-inhibitor complexes of LDH-A were solved with the loop in the open configuration [17-19].

Virtual screening and molecular modeling of protein interactions may assist in the identification of putative inhibitors in large compound libraries. However, such a modeling should take into account the mobility of the loop 96–111 that can affect binding efficiency. The objective of this study was to select an appropriate crystal structure of LDH-A, build the full-atom model on its basis, and verify the validity of the model for structure- based inhibitor screening and design.

## EXPERIMENTAL SECTION

Human LDH-A models have been constructed based on the crystal structure 1i10 [7] using the AmberTools 1.2 and Amber 10 packages (http://ambermd.org) [20]. Hydrogen atoms were added to the protein and ligands, and then the protein molecule was solvated in a TIP3P water box with a minimum distance of 12 A between the solute and the box edge (crystallographically resolved water molecules were retained). Chloride ions were added to charge neutrality. The energy minimization of the obtained system was performed using 2,500 steps of the steepest descent, followed by 2,500 steps of conjugate gradient, with positional restraints of 2 kcal/(mol × A^2^) on heavy atoms of protein and ligands. To describe the protein molecule, the *ff99SB *force field was employed [21]. The parameters for NADH were obtained from the AMBER parameter database [22]; for oxamate the parameters of the *GAFF *force field were used [23]. Water molecules and chloride ions were removed from the optimized structure to produce the Model 0 of LDH-A. Models 1 and 2 for docking simulation were obtained by removing oxamate and oxamate with NADH from Model 0, respectively.

The structures of pyruvate and known inhibitors of LDH were modeled using the ACD/ChemSketch 8.17 software [24]. Virtual screening for LDH-A inhibitors was performed among low-molecular-weight compounds from the Vitas-M library [25]. Compounds were protonated using OpenBabel 2.3.0 [26], and their 3D structures were generated with CORINA 3.4 [27]. Using the ACD/Spectrus DB 14.0 software [28], pyruvate and oxamate derivatives conforming to the Lipinski’s rule of five [29] were retrieved from the library.

Molecular docking into the active site of Models 1 and 2 with fixed amino acid coordinates was done using Lead Finder 1.1.15 [30]. The energy grid maps were computed for subunit A to overlap the binding site of oxamate (Model 1) or both binding sites of oxamate and NADH (Model 2). Minimum grid box was created around mentioned ligands (whose coordinates were derived from Model 0), and then the sides were moved away from the box center by a value of 6 A to include neighbourhood area. The energy of ligand binding was estimated accounting for van der Waals interactions, hydrogen bonding, electrostatics, and entropy changes due to desolvation and restriction of torsion angles. Docking runs were performed in the XP (extra precision) mode. RMS deviation values of the docking poses of inhibitors were calculated using reference coordinates obtained from the 1i10 and 4ajp structures (subunit A). An automated structural filtration was applied to modeled complexes to sort out ones exceeding the distance of 4.5 A between the carboxyl carbon of the ligand and the guanidinium carbon of Arg168.

Visualization, superimposition, and analysis of structures were performed using VMD 1.8.6 [31] and Swiss-PdbViewer 4.1.0 [32].

## RESULTS AND DISCUSSION

**Crystal structure selection**

**Fig. 1:**
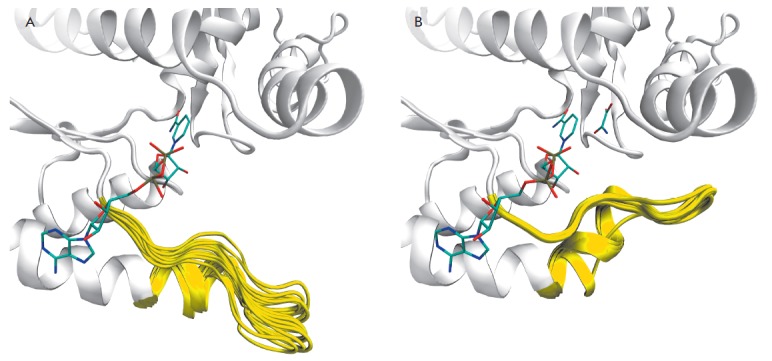
Open (*A*) and closed (*B*) conformations of human LDH-A according to X-ray crystallography. The loop 96–111 is colored yellow, and the positions of NADH and oxamate are colored by atom types. The ensemble of open conformations of the loop 96–111 was obtained by superimposition of separate subunits of the structures 4jnk (A, C, D), 4m49 (A–D), 4l4r (A, H), 4l4s (A, H), 4qo7 (A, C, D), 4qo8 (A, C, D) using the C^α^-atoms. The ensemble of closed conformations was generated by superimposing subunits of the structures 1i10 (A–C, E, F, H) and 4ajp (A–D)

The Protein Data Bank contains the following human LDH-A structures: the apo form (PDB ID 4l4r), the binary complex with NADH (4l4s), and complexes with inhibitors in the open (4jnk, 4m49, 4qo7, 4qo8) and closed (1i10, 4ajp) conformational states. To analyze the conformational space of the flexible loop 96–111, we superimposed individual subunits of these structures onto the subunit A of the 1i10 structure using Cα-atoms. The analysis shows that in the open conformation state the loop can be variously arranged, even within one tetramer (*Fig. 1A*). The superimposition of the subunits of the apo form 4l4r yielded a RMS deviation value of 2.09 A for the Cα-atoms of the loop. By contrast, in the closed state the loop appears to be stabilized in a unique configuration, with a slight shift in the subunit E of the 1i10 complex (*Fig. 1B*).

The conformational variability of the loop 96–111 in the open state complicates the choice of an appropriate structure for modeling and virtual screening. Recent work on LDH-A conformations advocated the use of ensemble docking, whereby the pose of a putative inhibitor is calculated for various protein structures, followed by an analysis of the generated complexes [33]. However, this approach processes large datasets and complicates the establishing criteria for the selection of potential inhibitors.

At the same time, the closed conformation of LDH-A favors structure-based inhibitor design due to the well-defined position of the loop 96–111. The prediction accuracy of closed-state models could be tested by docking substrates and known inhibitors. Therefore, the closed structures of human LDH-A 1i10 and 4ajp were of concern. The 1i10 structure at 2.30 A resolution is complexed with NADH and oxamate, and the 4ajp structure at 2.38 A resolution is complexed with the highly potent inhibitor 88N occupying the substrateand coenzyme-binding sites. The 1i10 structure was chosen for further modeling due to high resolution and the presence of coordinates of all residues within the tetramer.

**Construction of full-atom enzyme models**

Hydrogen atoms were added to the tetrameric LDH-A molecule derived from 1i10. The His192 residue was protonated on the Nδ1 and Nε2- atoms of the imidazole ring, whereas other ionizable residues in the active site (Arg98, Arg105, Arg168) were modeled in the standard charged form. Energy minimization of the solvated system was performed to adjust the positions of the added hydrogens. Following the removal of the bound ligands (NADH and/or oxamate) and water molecules, two LDH-A models were generated for docking simulations. Model 1 with NADH in the active site is designed for docking of compounds that compete with pyruvate, and Model 2 in its free form can be used for docking of compound competing with both pyruvate and NADH.

**Fig. 2:**
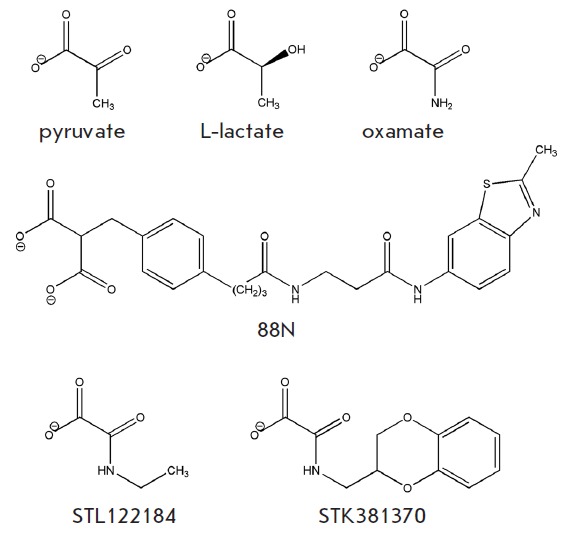
Substrates and inhibitors of human LDH-A

**Fig. 3:**
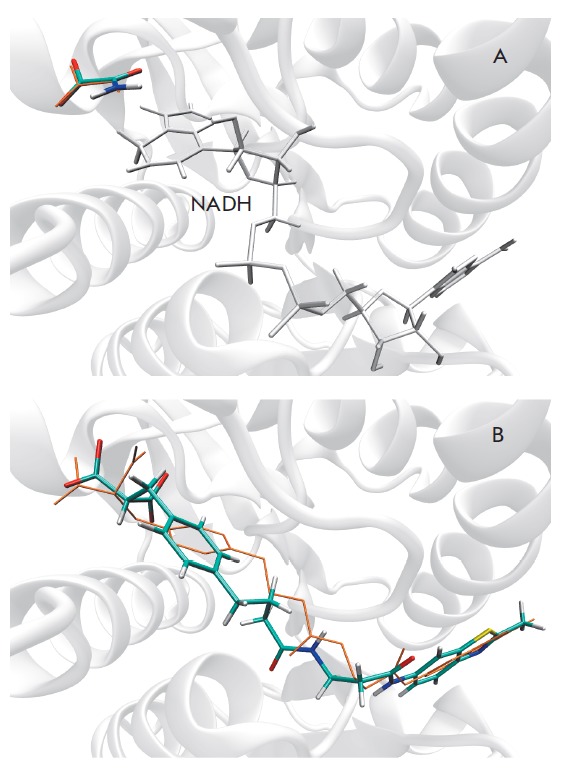
Poses of known inhibitors in the active site of human LDH-A as predicted by molecular docking. (*A*) The docking pose of oxamate in Model 1 containing NADH, Δ*G*^calc^ = –4.8 kcal/mol. (*B*) The docking pose of 88N in Model 2, Δ*G*^calc^ = –9.6 kcal/mol. Orange denotes the coordinates of compounds in the crystal structures 1i10 and 4ajp

The models were validated by docking human LDH-A inhibitors for which complex’s structure is known (*Fig. 2*). Oxamate, a substrate-like inhibitor, was docked into the active site of Model 1. The RMS deviation value of the predicted pose of oxamate from that of the 1i10 structure was 0.24 A (*Fig. 3A*). The docking simulations of substrate binding demonstrated that the pose of pyruvate is similar to that of oxamate, providing catalytically important interactions with Arg105, Arg168, His192, and the NADH nicotinamide ring. Docking of inhibitor 88N into the active site of Model 2 yielded a 1.65-A deviation from the crystallographic position in 4ajp (*Fig. 3B*). Known LDH-A inhibitors were correctly oriented in the model active site with a RMS deviation value of within 2 A with regard to the reference pose, which lends credence to the use of the docking algorithm applied.

**Accounting for loop 96–111 interactions**

Computer-aided screening with the generated LDH-A models should take into consideration interactions between substrates/inhibitors and the loop 96–111, which stabilized the closed conformation state. To identify hydrogen bonding and hydrophobic interactions that are formed upon loop closure, we compared structures of the apo form 4l4r and those of the enzyme-inhibitor complexes 1i10 and 4ajp.

In the complex 1i10, oxamate, a competitive analogue of pyruvate, forms hydrogen bonds with the Arg105 guanidinium group. This interaction with the loop is well known, since it plays an essential role in stabilizing the transition state during substrate conversion. There is also another hydrogen bond between the 3’-OH-group of the NADH nicotinamide and the backbone oxygen of Ala97; hydrophobic contact between the C2’- and C3’-atoms of the NADH nicotinamide and the side-chain C^β^-atom of Arg98; electrostatic interaction between the pyrophosphate of NADH and the guanidinium group of Arg98 see (*Table*). In the complex 4ajp, the carboxyl groups of inhibitor 88N interact with Arg105 in the same fashion as oxamate. In addition, one carboxyl group is hydrogen-bonded to the side chain of Gln99. The C21-atom of the methylene group and the C27-atom of the benzene ring form hydrophobic contact with the Cβ-atom of Arg98. Interestingly, no short-range interaction between the polar groups of the inhibitor and the guanidinium group of Arg98 is observed. The above-listed hydrogen bonds, electrostatic contacts, and hydrophobic interactions with the loop 96–111 are present in modeled complexes with pyruvate, oxamate, and inhibitor 88N and could be used as structural criteria for the selection of potential LDH-A inhibitors among screened compounds.

**Table:** Experimental groups

Interaction	Distance, Å	
	1i10	4ajp
Ala97:O ∙∙∙ NADH:O3’	2.88	
Arg98:CB ∙∙∙ NADH:C2’	3.71	
Arg98:CB ∙∙∙ NADH:C3’	3.56	
Arg98:NH1 ∙∙∙ NADH:P	4.0	
Arg105:NH2 ∙∙∙ OXM:O_carboxyl_	2.86	
Arg105:NE ∙∙∙ OXM:O_carbonyl_	2.93	
Arg98:CB ∙∙∙ 88N:C21		4.38
Arg98:CB ∙∙∙ 88N:C27		4.45
Gln99:NE2 ∙∙∙ 88N:Ocarboxyl2		2.72
Arg105:NH2 ∙∙∙ 88N:Ocarboxyl1		3.14
Arg105:NE ∙∙∙ 88N:Ocarboxyl2		3.04

**Virtual screening for inhibitors**

**Fig. 4:**
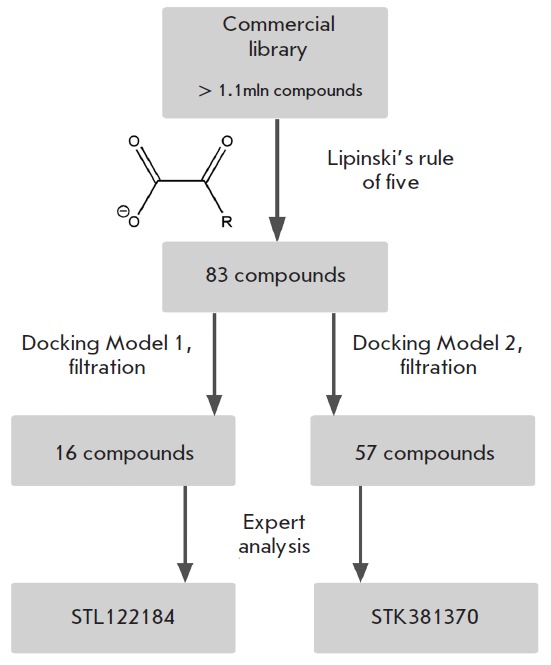
Flow-chart of virtual screening of a low-molecular-weight compound library against human LDH-A

The LDH-A models were evaluated by screening 83 pyruvate and oxamate derivatives (α-keto acids and their salts) retrieved from Vitas-M library using the Lipinski’s rule. This rule defines physico-chemical parameter ranges associated with drug-like compounds (molecular weight ≤500, log *P *≤5, hydrogen bond donors ≤5, hydrogen bond acceptors ≤10). When docked into the active site of Models 1 and 2, compounds were additionally filtered to sort out ones that do not form twin hydrogen bonds with Arg168 of the active site (this strong two-point interaction is involved in the binding of pyruvate and oxamate and should be common to substrate-like inhibitors). Via an expert analysis of modeled complexes, compounds capable of forming additional interactions with the protein (hydrogen bonds and hydrophobic contacts) were then selected. At this point, structural criteria for potential LDH-A inhibitor were at least one (for Model 1) or two (for Model 2) interactions with the loop 96–111 listed in Table. Two compounds were eventually selected: STL122184 (Δ*G*^calc^ = –4.9 kcal/mol) and STK381370 (Δ*G*^calc^ = –7.9 kcal/mol) for Model 1 and 2, respectively ( *Fig. 2*, *Fig. 4*).

**Fig. 5:**
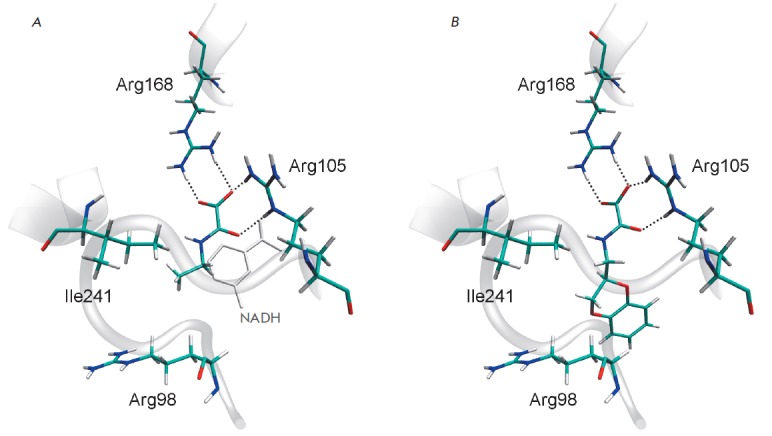
Positions of potential inhibitors in the active site of human LDH-A revealed by virtual screening of a commercial compound library. (*A*) The docking pose of STL122184 in Model 1. (*B) *The docking pose of STK381370 in Model 2. The Arg98 and Arg105 residues of the mobile loop 96-111 are shown

STL122184 (N-ethyloxamic acid) was recently shown to compete with pyruvate for binding to LDH-A from mouse skeletal muscle (*K*i = 140 µKM) [34]. When docked, STL122184 forms twin hydrogen-bonded contacts with the guanidine group of Arg168, hydrogen bonds with Arg105, and forms a hydrophobic contact between the ethyl moiety and the Ile241 side chain (*Fig. 5A*). Interestingly, STK499896 (N-isopropyloxamic acid) and STK501930 (N-propyloxamic acid), close structural analogs of STL122184, were discarded because of no hydrogen bonding with Arg168 and an unfavourable interaction of hydrophobic substituent with the backbone of Thr247, respectively. Experimental testing of these compounds against mouse LDH-A also showed low inhibitory potencies [34, 35].

STK381370 remains yet to be tested for inhibitory activity. This putative inhibitor of LDH-A forms all the necessary interactions listed for Model 2: twin hydrogen- bonded contact with Arg168, hydrogen bonds with Arg105, and a hydrophobic contact with the side chain of Arg98 (*Fig. 5B*). In addition, the polycyclic moiety of STK381370 may form hydrogen bond with the sidechain of Asn137 and a hydrophobic contact with Val30.

## CONCLUSIONS

The flexibility of the loop 96–111, which forms part of the active site of human LDH-A, dramatically contributes to substrate binding. An analysis of X-ray crystal structures revealed the conformational variability of the loop in the open state. After LDH-A proceeds to the closed state, the loop conformation is stabilized by hydrogen bonds and hydrophobic contacts formed by Ala97, Arg98, Gln99, and Arg105 with bound substrates and inhibitors.

On the basis of the crystal tetrameric structure 1i10, we constructed full-atom models of human LDH-A (in complex with NADH and in the apo form) that showed promise in virtual screening of a low-molecular-weight compound library. The established criteria for the selection of putative inhibitors were hydrogen bonds and hydrophobic contacts with the loop 96–111. They enabled us to identify a potential inhibitor, STK381370, whose docking pose was stabilized through additional interactions with Arg105 and Arg98.

## Abbreviations

LDH: lactate dehydrogenase
LDH-A: lactate dehydrogenase isoform A
88N: inhibitor name presented in the 4ajp crystal structure
